# Arsenic causing gallbladder cancer disease in Bihar

**DOI:** 10.1038/s41598-023-30898-0

**Published:** 2023-03-14

**Authors:** Arun Kumar, Mohammad Ali, Vivek Raj, Arti Kumari, Mahesh Rachamalla, Som Niyogi, Dhruv Kumar, Ashok Sharma, Amit Saxena, Ghanish Panjawani, Preeti Jain, Ajay Vidyarthi, Navin Kumar, Mukesh Kumar, Pintoo Kumar Niraj, Md. Samiur Rahman, Akhouri Bishwapriya, Ranjit Kumar, Maiko Sakamoto, Santosh Kumar, Manisha Singh, Ashok Kumar Ghosh

**Affiliations:** 1 Mahavir Cancer Sansthan and Research Centre, Patna, Bihar 801505 India; 2 Department of Biotechnology, Patna Women’s College, Patna, Bihar India; 3 University of Saskatchewan, Saskatoon, SK S7N 5E2 Canada; 4 School of Health Sciences & Technology, UPES University, Dehradun, Uttrakhand India; 5 All India Institute of Medical Sciences, New Delhi, India; 6 Centre for Development of Advanced Computing (C-DAC), Pune, Maharashtra India; 7 Geological Survey of India, Patna, Bihar India; 8 Central University of Himachal Pradesh, Kangra, India; 9 University of Tokyo, Kashiwa, Japan; 10 Delft University of Technology, Delft, The Netherlands

**Keywords:** Environmental impact, Cancer, Environmental sciences, Oncology

## Abstract

In recent times Gallbladder cancer (GBC) incidences increased many folds in India and are being reported from arsenic hotspots identified in Bihar. The study aims to establish association between arsenic exposure and gallbladder carcinogenesis. In the present study, n = 200 were control volunteers and n = 152 confirmed gallbladder cancer cases. The studied GBC patient’s biological samples-gallbladder tissue, gallbladder stone, bile, blood and hair samples were collected for arsenic estimation. Moreover, n = 512 gallbladder cancer patients blood samples were also evaluated for the presence of arsenic to understand exposure level in the population. A significantly high arsenic concentration (*p* < 0.05) was detected in the blood samples with maximum concentration 389 µg/L in GBC cases in comparison to control. Similarly, in the gallbladder cancer patients, there was significantly high arsenic concentration observed in gallbladder tissue with highest concentration of 2166 µg/kg, in gallbladder stones 635 µg/kg, in bile samples 483 µg/L and in hair samples 6980 µg/kg respectively. Moreover, the n = 512 gallbladder cancer patient’s blood samples study revealed very significant arsenic concentration in the population of Bihar with maximum arsenic concentration as 746 µg/L. The raised arsenic concentration in the gallbladder cancer patients’ biological samples—gallbladder tissue, gallbladder stone, bile, blood, and hair samples was significantly very high in the arsenic exposed area. The study denotes that the gallbladder disease burden is very high in the arsenic exposed area of Bihar. The findings do provide a strong link between arsenic contamination and increased gallbladder carcinogenesis.

## Introduction

Gallbladder cancer (GBC) is one of the rarest biliary tract malignancies with high mortality rate with relatively less survivability of 5 years^1,2^. Globally in year 2020, out of total cancer cases of 19,292,789, new GBC cases reported in the year was 115,949 while number of deceased cases were 84,695^3^. India accounts for 10% of global GBC cases, about one million new cancer cases every year with mortality rate as high as 33% every year^4^. According to Indian Council of Medical Research (ICMR), it is estimated that by the year 2025, there will be fivefold increase in cancer incidences in India, in which there will be 2.8-fold increase due to tobacco while 2.2-fold increase due to ageing. Such a drastic increase in cancer cases is more than the double which is predicted for the developed countries like USA^5–7^.

GBC has a very unusual geographical distribution with high incidences seen in pockets as high incidences are seen in Chile, India, Japan, Poland, Israel, Bolivia, Thailand, South Korea etc.^1,8–10^. In India, the highest distribution of GBC has been detected in the states like Uttar Pradesh, Bihar, Orissa, West Bengal, Assam and Delhi (National Capital Territory)^11–14^. The cancer is two folds more common in women in comparison to men^15^. Population based data reveals that the incidences of this disease is very high in the northern cities of India (22 per 100,000) and low (0–0.7 per 100,000 women) in southern India^16–19^. The present institute caters the greatest number of cancer patients from the state of Bihar for the diagnosis and the treatment. Among the total cancer patients, 8.3% and 16.9% are GBC cases in male and female, respectively^5^. GBC patients exhibit various clinical symptoms, including benign biliary disease on one hand and incurable malignant on other hand^20–22^. The etiology of gallbladder cancer apart from the few reasons has been a mystery, however the interplay of gallstones, genetic susceptibility, changes in the lifestyle factors and infections lead to progression of the cancer disease^23,24^.

Environmental pollutants in the recent times have become serious health hazards to the population. Due to ever increasing anthropogenic activities the level of the pollution effect has increased manyfold leading to eruption of the diseases in the population^25^. Human exposure to arsenic a non-essential metalloid, has increased in recent times due to geogenic changes and anthropogenic activities, which has caused severe health hazards in populations around the world^26–28^. The inorganic arsenic when enters the human body is metabolized to organic arsenic (Monomethylarsonous acid-MMA(III), dimethylarsinic acid-DMA (V) and trimethylarsine TMA) which is still a toxic carcinogen. These organic compounds bind with DNA molecules which can influence transcription and translation leading to cancer. However, it is eliminated by the body as a metabolic waste via kidney. It also gets deposited in the keratin containing tissues like hair, nail and skin^29^ Arsenic is known to be a carcinogen (as carcinogen category-I) in all the forms—arsenic trioxide, arsenic pentoxide, arsenous acids, arsenic acids and their salts (arsenites and arsenates)^30–34^. Humans are exposed to this inorganic arsenic mainly by two ways—consumption of arsenic contaminated water and food^35–38^.

Arsenic is known to cause cancer of skin, bladder, lungs, kidney, liver and prostate^39–41^. Arsenic causes cellular toxicity by inducing oxidative stress (ROS) which in turn leads to genotoxicity^42^. The continuous exposure of arsenic creates a very high oxidative stress that eventually leads to the appearance of symptoms like arsenicosis, skin lesions, black foot disease, vascular disease, hormonal imbalance and cancer^43–45^. Due to its physical characters (no odour, colour or flavour), arsenic exposure is often unnoticed until people develop some arsenicosis symptoms due to drinking of arsenic contaminated water. The World Health Organisation (WHO) and the U.S Environmental Protection Agency (USEPA) have recommended a threshold of 10 µg/L for inorganic arsenic concentration in drinking water. Unfortunately, millions of people are exposed to much higher toxic levels of arsenic and some populations are unaware of its ill effects, which in long term is causing the development of malignancies. Various molecular pathways, which has been deciphered in recent times, shows the progression of the disease by arsenic biotransformation process, that alters the methylation pattern, which is thought to play a key role in its carcinogenicity^46–51^.

In the Gangetic plains of Bihar, a high percentage of population suffers from arsenic poisoning due to arsenic contamination of groundwater. The long duration exposure to this arsenic has magnified the toxicity by many folds causing deadly disease like cancer in human beings^52^. Recently, the gallbladder cancer disease burden in these areas have increased many times and the situation is catastrophic^53^. Hence, there is an urgent need to investigate the relationship between arsenic exposure and GBC in the local populace. There has not been any significant study which confirms the linkage between arsenic contamination in groundwater and increased incidence of gallbladder cancer till date. The present study throws new light on the association between gallbladder carcinogenesis and arsenic poisoning in the Gangetic plains of Bihar through the novel pathway.

## Materials and methods

### Ethical approval

Ethical approval was obtained from the Institutional Ethics Committee of the Indian Council of Medical Research (ICMR)-Rajendra Memorial Research Institute and Medical Sciences, Patna, Bihar, India with IEC Ref No. 07/RMRI/EC/2019, dated 20/06/2019. All the patients were informed about the research study and written informed consent were obtained prior to their inclusion in the study.

### Study group

For the control study, volunteers (non-diseased subjects) were selected, while the gallbladder cancer patients who were undergoing gallbladder surgeries at the parent cancer institute with the assistance of the institute’s Surgical Oncology Department were selected as diseased subjects. For the present study, volunteers were divided into confirmed Gallbladder Cancer cases (n = 152) and control cases (n = 200) after obtaining their consents. Moreover, n = 512 gallbladder cancer patients (of institute itself) blood samples were evaluated for arsenic contamination to understand the exposure level in the population. The demographic and clinical profiles of volunteers were obtained from the central records of the institute for the baseline information. The sample size numbers have been calculated through the formula and as per the previous studies carried out by our team^54^.

### Collection of samples

After the surgery, all the biological samples—blood, gallbladder tissue, gallbladder stones, gallbladder bile and hair samples of the volunteers of gallbladder benign as well as the GBC cases were collected. The samples were subsequently stored in − 80 °C deep freezer prior to the analysis.

### Analytical quality control

For the analytical quality control, the blanks, procedural blanks, replicates, standards, correlation coefficient and detection limits were maintained for arsenic analysis in the atomic absorption spectrophotometer. The known standard concentration of arsenic was prepared from the standard arsenic stock solution (1000 µg/L) procured from PerkinElmer (CAS# As 7440-38-2; Lot# 20-85 ASX1; PE# N9300102), Singapore. The arsenic detection limit for blood was 0.05 µg/L in the employed method. The cancer patient’s tissues, gallstones and bile samples were analysed for the first time in this part of the region, the protocol of National Institute for Occupational Safety and Health (NIOSH, 1994) was utilised^55^.

### Analysis of the samples through Atomic Absorption Spectrophotometer

1. **Analysis of Gallbladder bile and blood samples:** After the collection, all the tissue samples were digested using concentrated HNO_3_ (Merck analytical grade 69%) on hot plate under fume-hood and arsenic was estimated as per the protocol of National Institute for Occupational Safety and Health (NIOSH, 1994) by Graphite Furnace Atomic Absorption Spectrophotometer (GF-AAS) (Pinnacle 900T, Perkin Elmer, Singapore) at the studied institute. The blood and gallbladder bile samples were taken as 0.5 ml aliquots in 30 ml conical flask and 5 ml HNO_3_ was added and left for overnight reaction. The samples were then digested on hotplate at 90–120 °C until the sample reached to 3 ml. Then 5 ml mixture of HNO_3_:HClO_4_ (6:1) (HClO_4_—Merck analytical grade 70%) was added to the solution and again re-digested on hotplate until the volume reached to about 2 ml. Final volume was adjusted to 10 ml with 1% HNO_3._ The solutions were filtered through Whatman filter paper no.41 and analysed through GF-AAS.
2. **Analysis of Gallbladder tissue and Gallstone samples:** The gallbladder tissue and gallbladder stone samples were taken as 0.5 g mass in the conical flask and 5 ml of Conc. HNO_3_ was then added to the flask and left for overnight reaction. The samples were digested next day on hotplate at 60 °C for 2 h. After that, the samples were cooled and then added with 2 ml of HClO_4_ and then re-digested at 90–120 °C for 5–10 min until the white fume of HClO_4_ is emitted. The solution was cooled and diluted with 15 ml with deionized water and filtered with Whatman filter paper no.41 and analysed by GF-AAS.
3. **Analysis of hair samples:** For hair samples analysis, 0.120 g of hair samples were weighed and washed in 15 ml of 0.1% SDS solution (Sigma-Aldrich) and sonicated for 10 min. The solution was decanted and washed with ultrapure distilled water. Then the hair samples, were placed in 15 ml of high-grade acetone solution and sonicated for 10 min. The solution was decanted and rinsed with ultrapure distilled water three times. The hair samples were dried at 40 °C for 5 min in the oven and then 10 ml of HNO_3_ was added and covered with watch glass and digested on hotplate 90–120 °C. When the solution was reduced to 3 ml, 1 ml of H_2_O_2_ was added and it was re-digested at the previous temperature until the bubbles came out and the solution was reduced to 2.5 ml. Then the heating was stopped, and the solution was rinsed with 1% HNO_3_ and adjusted to a final volume of 10 ml with ultrapure distilled water. The sample was then filtered through Whatman filter paper no.41 and analysed through GF-AAS.

### Molecular Docking analysis of Arsenic compounds with haemoglobin, cysteine and taurine

1. **Retrieval and preparation of target protein and arsenic compounds**: The 3D-Crystal structure of human haemoglobin (PDB ID: 4HHB) was downloaded from Protein Data Bank (https://www.rcsb.org/structure/4HHB) in pdb format^56^. Human haemoglobin consists of 4 subunits (2 alpha subunit, chain A and chain C; 2 beta subunits, chain B and chain D). The structure was prepared for docking in Chimera X 1.1^57^. Haemoglobin alpha subunit chain A and beta subunit chain B was used for docking in separate experiments. Arsenous acid (Trihydroxy-arsenite (III), As(OH)_3_, PubChem ID 545) 2-D structure was converted to 3D coordinates by MolView web based open-source application and was downloaded as mol file. The ligand molecule was also prepared in Chimera X 1.1 and saved as pdb. The 3D structure of Cysteine (PubChem CID: 5862) and taurine (PubChem CID: 1123) was downloaded from PubChem database as sdf file. These two molecules were also prepared and saved as pdb for further study.
2. **Docking analysis:** Docking was performed by HDOCK webserver^58^. The HDOCK server is to predict the binding complexes between two molecules using a hybrid docking strategy. Firstly, docking was performed between chain A of human haemoglobin and arsenous acid. Haemoglobin was uploaded as receptor and arsenous acid served as ligand molecule. In second experiment, human haemoglobin chain B served as receptor and docking was performed with arsenous acid ligand molecule. Similarly, cysteine as receptor was docked with ligand arsenous acid as a separate experiment and taurine as receptor was docked with arsenous acid at HDOCK webserver as another experiment to understand the interacting residues between two molecules. The results were visualised in Chimera X 1.1 and Pymol Viewer^59^.
  **GIS Mapping:** The data of arsenic concentration in blood samples of the GBC patients (n = 512) were taken as input in ArcGIS Version 10.5.1 for spatial analysis and visualization. The location data of collected samples of the GBC patients were plotted and extracted as a GIS map layer for visualization. The study was conducted to understand the spatial pattern of the GBC patients geographically in the arsenic exposed area, and the resultant distribution map result is shown in Fig. 2. The arsenic concentration measured in the five biological samples (tissue, stone, bile, blood and hair) of the GBC patients from the arsenic exposed area were also analyzed in the ArcGIS environment. The output of five biological samples data, location of the GBC patients and the exposure rate pattern is shown in Fig. 6. The software used in the map layer generation was ArcGIS Version 10.5.1.

### Statistical analysis

Data were analysed with statistical software (GraphPad Prism 5) and values were expressed as Mean ± SEM. Differences between the groups were statistically analysed by one-way analysis of variance (ANOVA) followed by the Dunnett’s test for comparison among study groups. The scatter plot graphs were designed using another statistical software SPSS-16.0 utilising the linear regression model as earlier used^60^.

### Principal component analysis

Principal Component Analysis (PCA) was applied to construct a holistic evaluation index from the 152 patients' data. The used variables were arsenic concentrations in blood samples of gallbladder benign and gallbladder blood (BLOOD), gallbladder tissue (TISSUE), gallbladder stones (STONE), gallbladder bile (BILE), and hair (HAIR) samples. All the variables were standardized. The R function prcomp was used for computation^61^.

### Ethics approval and consent to participate

The present research work was carried in accordance with the Declaration of Helsinki and the ethical approval were obtained from the Institutional Ethics Committee of the Indian Council of Medical Research (ICMR)-Rajendra Memorial Research Institute and Medical Sciences, Patna, Bihar, India with IEC Ref No. 07/RMRI/EC/2019, dated 20/06/2019. All the patients were informed about the research study and written informed consent were obtained prior to their inclusion in the study.

## Results

In the present study, all the collected samples were analysed –

1. **Gender wise patients in control versus gallbladder cancer patients:** Out of total n = 200 control samples, there were n=76 male and n=124 female patients. Among the confirmed n=152 GBCs, there were n=37 male and n=115 female patients.
2. **Age group of patients in control and gallbladder cancer patients: **Out of total n = 200 control cases, the maximum age observed was 58 years, while the minimum was 21 years. Out of n=152 confirmed GBCs, the maximum age was 90 years, while the minimum was 25 years.
3. **Stagewise distribution of gallbladder cancer patients:** Out of n = 152 confirmed GBCs, there were only n = 3 in stage-I disease (1.9%), whereas n = 11 patients were in stage-II disease (7.2%), n = 15 were in stage-III disease (9.8%), and n = 123 patients in stage-IV (80.9%) disease.
4. **Arsenic concentration in tissue samples of gallbladder cancer patients:** Out of total n = 152 confirmed GBCs cases, the maximum arsenic concentration was 2166 µg/kg, while the minimum was 0.67 µg/kg (Table 1).
5. **Arsenic concentration in gallstone samples of gallbladder cancer patients:** Out of total n = 152 confirmed GBC patient’s the maximum arsenic concentration was 634.9 µg/kg, while the minimum was 0.03 µg/kg (Table 1).
6. **Arsenic concentration in bile samples of gallbladder cancer patients:** Out of n = 152 confirmed GBCs, the maximum arsenic concentration was 483.2 µg/L, while the minimum was 0.17 µg/L (Table 1).
7. **Arsenic concentration in blood samples in control versus gallbladder cancer patients:** Out of the total n = 200 control volunteers, the maximum arsenic concentration observed was 11.7 µg/L, while minimum was zero µg/L. Out of n=152 confirmed GBCs cases, the maximum arsenic concentration was 389.3 µg/L, while the minimum was 0.2 µg/L (Table 1).
8. **Arsenic concentration in hair samples of Control versus gallbladder cancer patients:** Out of the total n = 200 control volunteers, the maximum arsenic concentration observed was 14.4 µg/kg, while the minimum was zero µg/kg. Out of n = 152 confirmed GBC cases, the maximum arsenic concentration was 6980.4 µg/kg, while the minimum was 0.2 µg/kg (Table 1).

**Table 1:** Arsenic concentration in biological samples of gallbladder cancer patients

Biological samples studied	Arsenic concentration (mean ± S.E)	Maximum arsenic concentration	Minimum arsenic concentration	Lower 95% CI of mean	*P* value
GBC Patient’s Tissue Arsenic Concentration (µg/Kg) (n = 152)	**340.6 ± 31.98**	2166	0.6700	277.4	< 0.0001
GBC Patient’s Gallstone Arsenic Concentration (µg/Kg) (n = 152)	**78.41 ± 8.311**	634.9	0.0300	61.99	< 0.0001
GBC Patient’s Bile Arsenic Concentration (µg/L) (n = 152)	**59.10 ± 6.554**	483.2	0.0230	46.15	< 0.0001
GBC Patient’s Blood Arsenic Concentration (µg/L) (n = 152)	**52.28 ± 5.504**	389.3	0.2000	41.41	< 0.0001
GBC Patient’s Hair Arsenic Concentration (µg/Kg) (n = 152)	**649.7 ± 93.88**	6980.1	0.2000	464.2	< 0.001
Control Subject’s Blood Arsenic concentration (µg/L) (n = 200)	**0.8133 ± 0.1670**	11.70	0	0.4840	< 0.001
Control Subject’s Hair Arsenic Concentration (µg/Kg) (n = 200)	**1.541 ± 0.2351**	14.40	0	1.077	< 0.0001

Data are represented as Mean ± Standard Error using (ANOVA-Dunnett’s Test, *P* < 0.05).

Significant values are in bold.
9. **Correlation Coefficient between gallbladder cancer patient’s tissue and bile:** There was a significant positive correlation showing an increasing trend between the arsenic concentrations in between the gallbladder tissue, gallstone, bile, blood and hair samples of confirmed GBC patients (Fig. 1).

**Figure 1:**
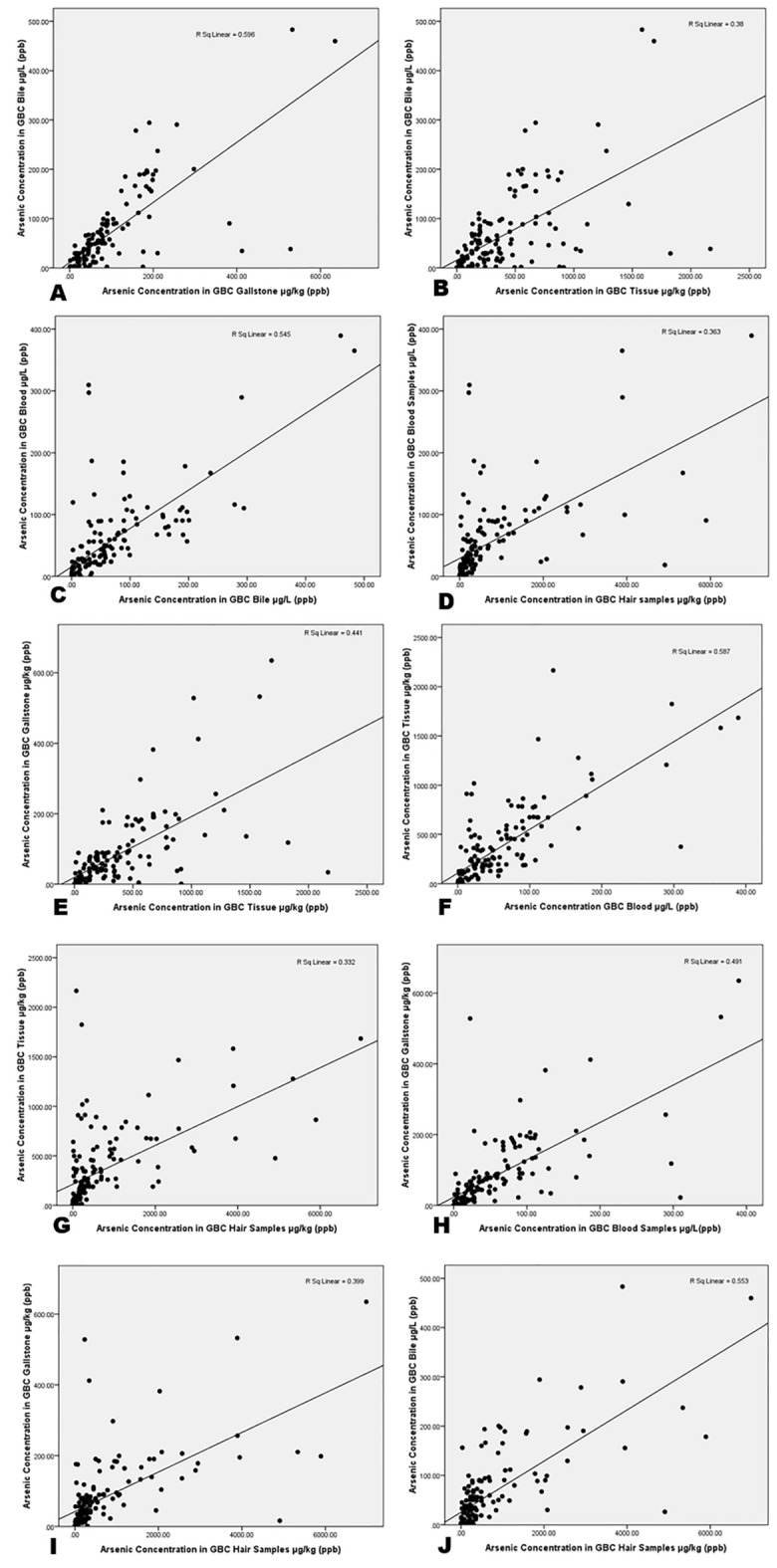
The scatterplot graphs of gallbladder cancer patients (**A**) GBC bile and gallstone (r = 0.596 & *p* < 0.05) (**B**) GBC bile and tissue (r = 0.38 & *p* < 0.05) (**C**) GBC bile and blood (r = 0.545 & *p* < 0.05) (**D**) GBC blood and hair(r = 0.363 & *p* < 0.05) (**E**) GBC gallstone and tissue (r = 0.441 & *p* < 0.05) (**F**) GBC blood and tissue (r = 0.587 & *p* < 0.05) (**G**) GBC hair and tissue (r = 0.332 & *p* < 0.05) (**H**) GBC gallstone and blood (r = 0.491 & *p* < 0.05) (I) GBC gallstone and hair (r = 0.399 & *p* < 0.05) (**J**) GBC bile and hair (r = 0.553 & *p* < 0.05).
10. **Arsenic Concentration in blood samples of n = 512 gallbladder cancer patients:** The blood arsenic concentration in the n = 512 gallbladder cancer patients showed significant raised levels in n = 274 (53.6%) patients having concentration more than 15 µg/L, while n = 238 (46.4%) had concentration less than 15 µg/L. The maximum arsenic concentration observed was 746.43 µg/L. In the study, n = 338 were female GBC patients while n = 174 were male GBC patients (Fig. 2).

**Figure 2:**
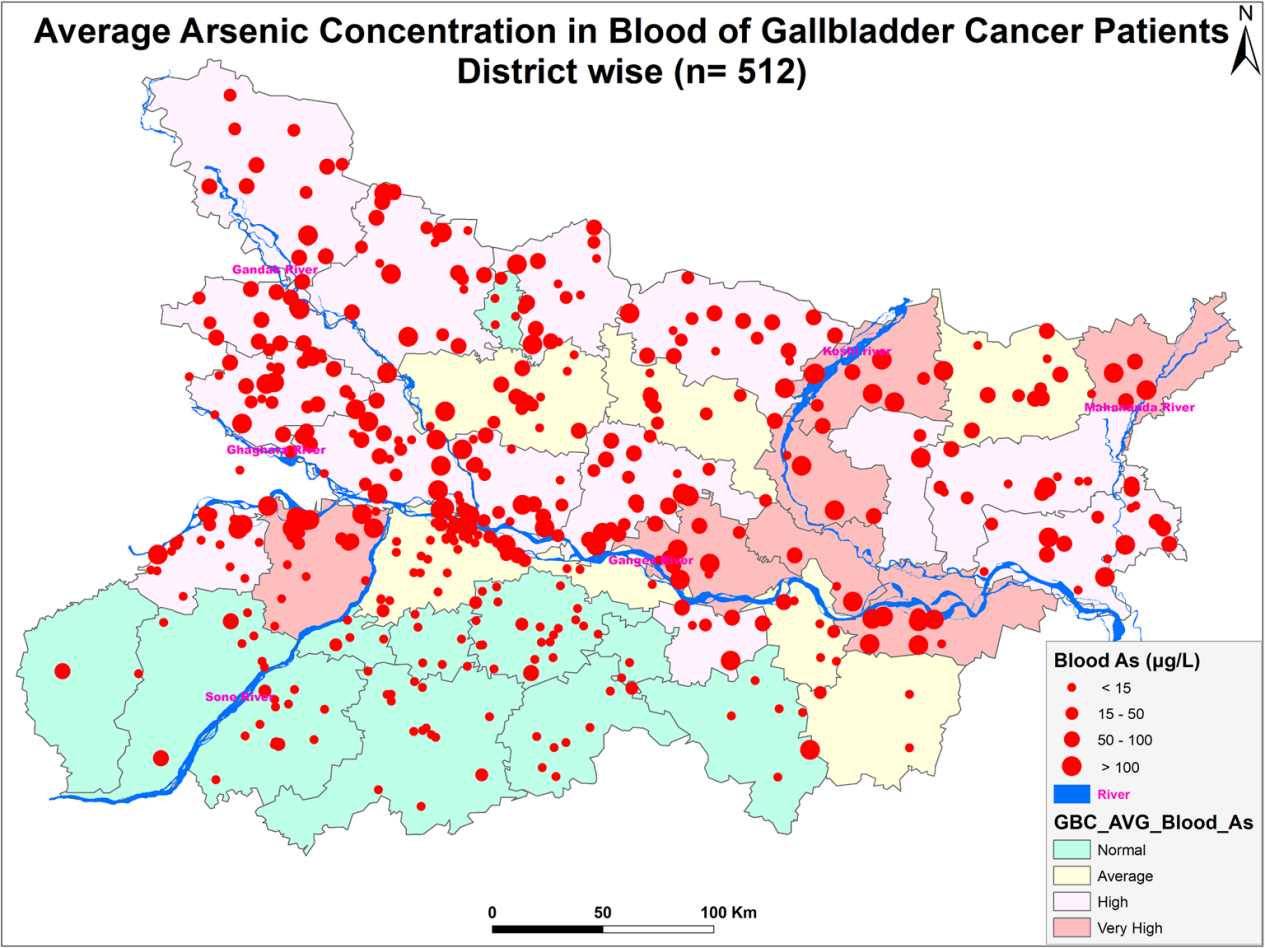
Figure showing distribution of GBC cancer patients (n = 512) (Arc-Gis-10.5.1).
11. **Principal Component Analysis (PCA):** (Table 2) shows the loadings and variances for each principal component.

**Table 2.** PCA Loadings applied to arsenic concentrations in biological samples.

	PC1	PC2	PC3	PC4	PC5
TISSUE	0.853	0.399	− 0.230	0.199	− 0.145
BLOOD	0.892	0.268	− 0.025	− 0.314	0.182
HAIR	0.830	− 0.443	− 0.315	0.075	0.105
STONE	0.885	− 0.013	0.376	0.244	0.124
BILE	0.907	− 0.221	0.163	− 0.185	− 0.259
Eigen value	**3.82**	**0.476**	**0.320**	**0.238**	**0.148**
Proportion of variance (%)	**76.4**	**9.53**	**6.41**	**4.76**	**2.96**

Significant values are in bold.
  Only the first principal component (PC) had the eigenvalue larger than 1 and accounts for 76.4 % of the total variances. Thus, we picked only the PC 1 to construct a holistic evaluation index of the arsenic accumulation in biological samples. Equation (1) shows the constructed index, which is a formula to calculate the PC1 scores for each patient where the coefficients are based on the estimated eigenvector information. Interestingly, the coefficients (and loadings shown in Table 1) are more or less equivalent among the five variables. This means that each standardized variable similarly contributes to maximize the holistic index variance and suggests the heterogeneity in the arsenic accumulation process. (Fig. 3a) shows the PC1 scores ordered from the highest to the lowest with the cancer stage information (stage 1–4 represent the cancer stage, whereas stage 0 means non-cancer onset). (Fig. 3b-f) show the arsenic concentration for the biological samples ordered from the highest to the lowest for each sample.
  1 $$\begin{aligned} {\text{Holistic}}\;{\text{evaluation}}\;{\text{index}} & = 0.436 \times {\text{TISSUE}} + 0.457 \times {\text{BLOOD}} + 0.425 \times {\text{HAIR}} \\ & \quad + 0.453 \times {\text{STONE}} + 0.464 \times {\text{BILE}} \\ \end{aligned}$$

**Figure 3:**
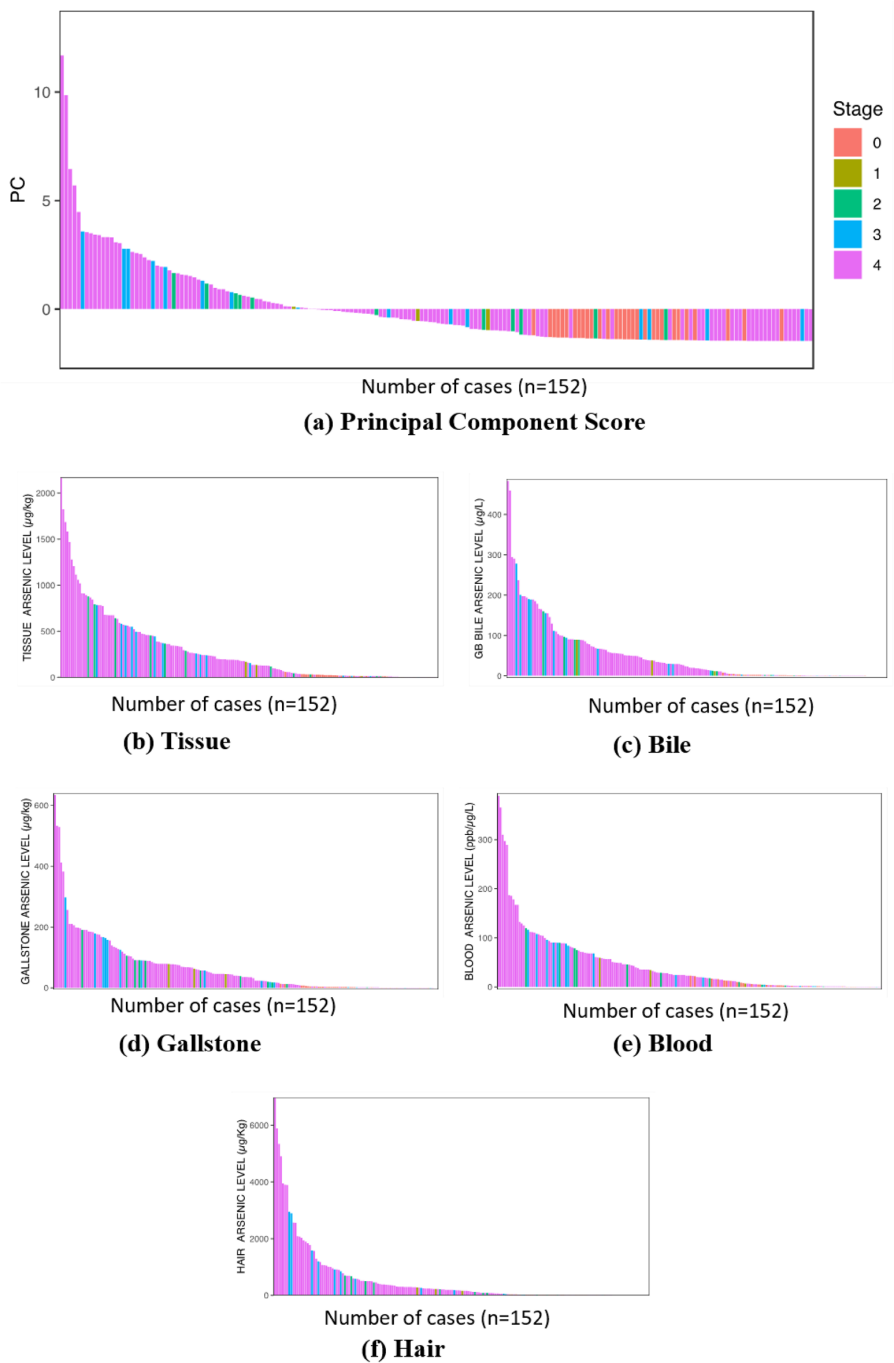
The bar graph on (**a**) PC scores and (**b**–**c**) arsenic concentration for each biological sample with the cancer stage information.
  Figure 3b-f shows that the gallbladder cancer patients tended to have higher arsenic accumulations for each biological sample than non-cancer patients. Figure 3a made the accumulation characteristics more visible and demonstrates that a significant number of cancer patients are found on the right edge of the bar graph. Their overall arsenic accumulation in their bodies is low, so perhaps they have not been intensively exposed to arsenic in their daily lives. Therefore, in the case of these patients, the development of gallbladder cancer is less likely due to the arsenic intake but their inherent vulnerability to the cancer prevalence or other environmental factors.
12. **Proposed Novel Pathway of Gallbladder carcinogenesis:** The present study proposes following pathway for the gallbladder carcinogenesis caused due to arsenic poisoning. Since, humans in the Gangetic plains of Bihar are long term exposed to arsenic poisoning, hence their susceptibility to cause disease is very significant. The mode of exposure causing disease burden has been proposed below (Fig. 4).

**Figure 4:**
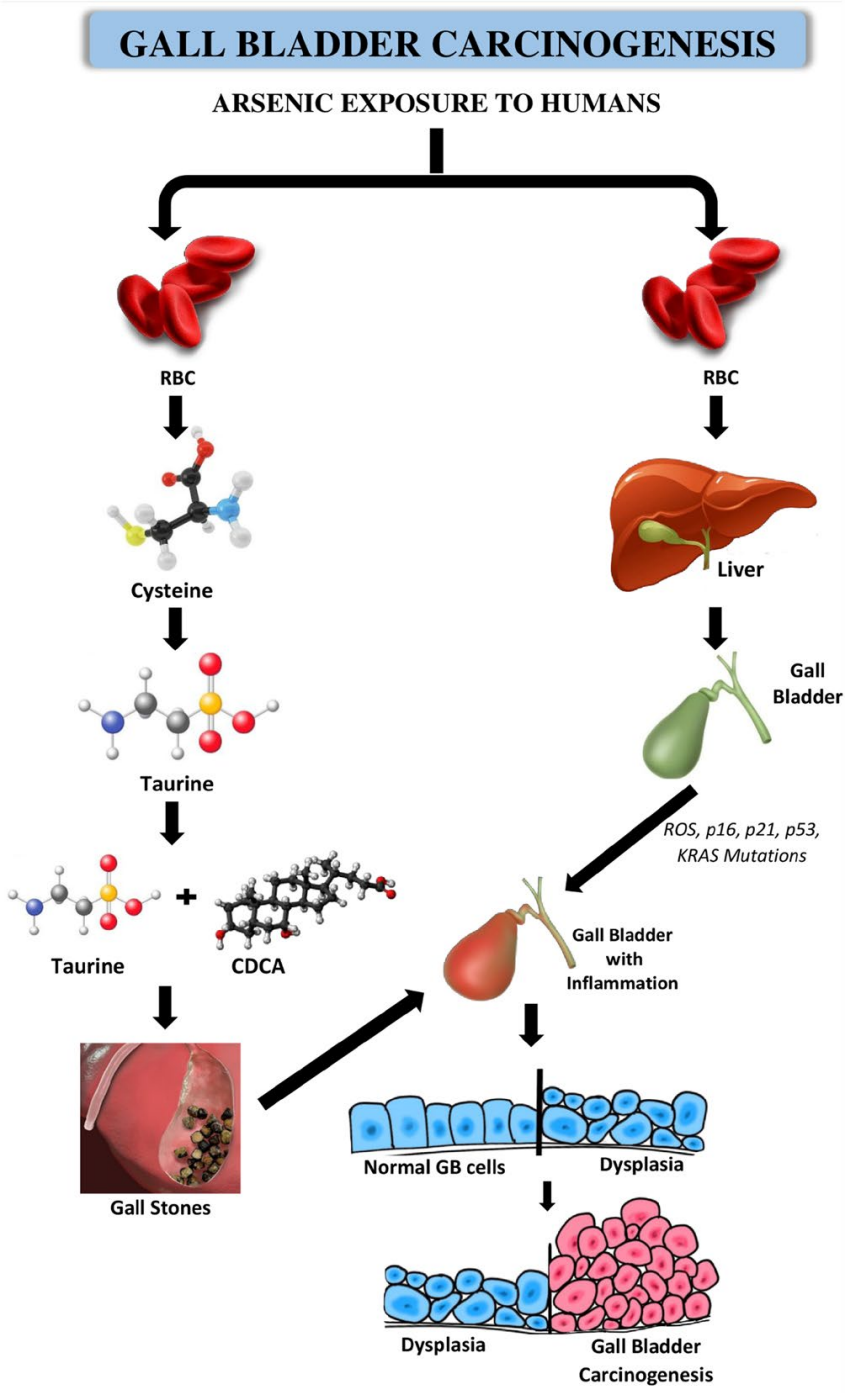
Schematic diagram showing the etiology of Gallbladder carcinogenesis.

**Figure 5:**
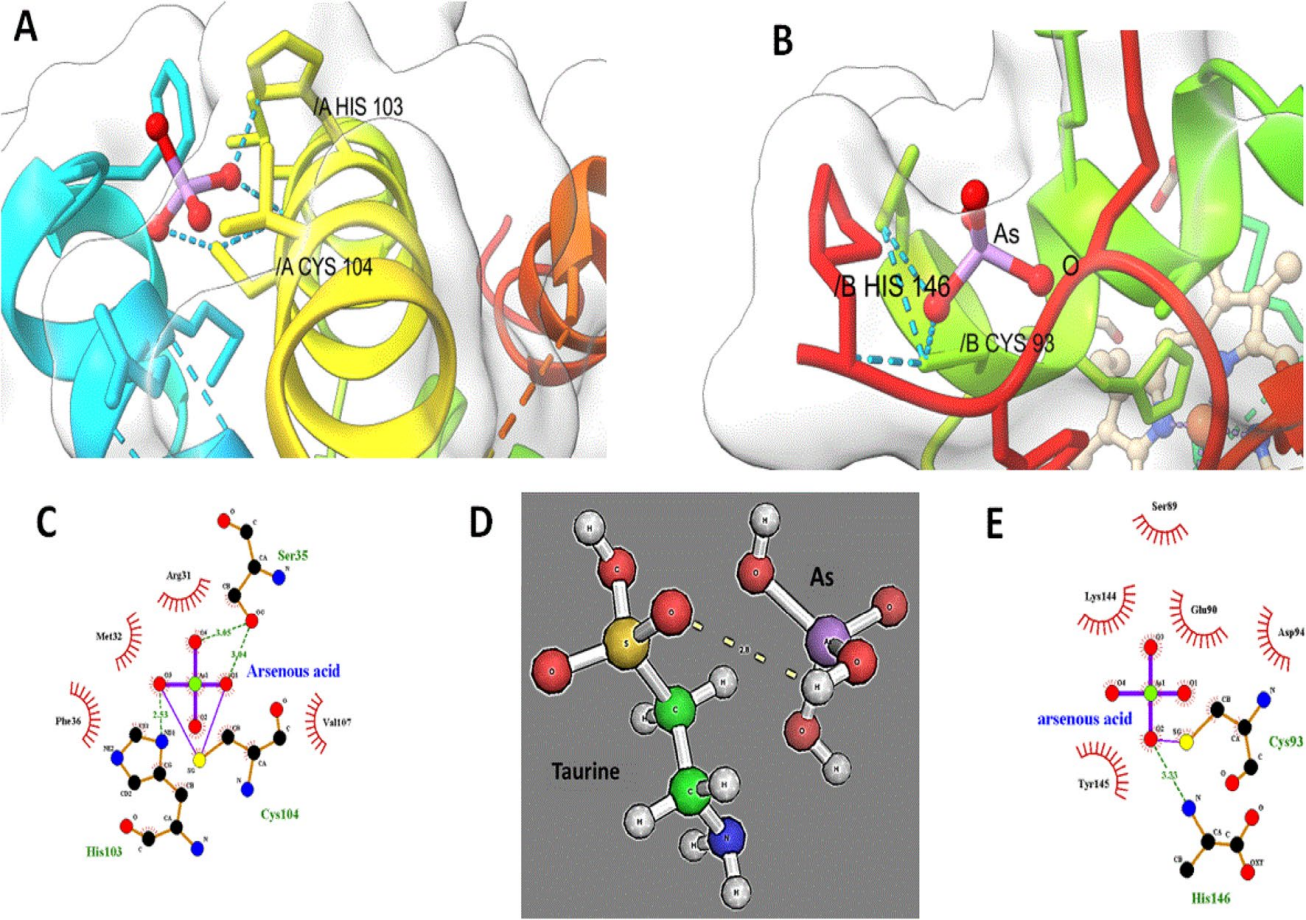
(**A**) Docking structure of Human Haemoglobin (PDB ID: 4HHB, bound with As III (Arsenous acid, PubChem ID: 545) at Cys 104 residue in chain A. (**B**) Docking structure of Human Haemoglobin (PDB ID: 4HHB, bound with As III (Arsenous acid, PubChem ID: 545) at Cys 93 residue in chain B (**C**) Hydrophilic and Hydrophobic interaction of the As III (arsenous acid) bound with chain A of human haemoglobin. (**D**) Interaction of taurine with Arsenic (**E**) Hydrophilic and Hydrophobic interaction of the As III (arsenous acid) bound with chain B of human haemoglobin.

  12. Molecular Docking to Establish the Novel Pathway: Association between arsenic and its conjugation with sulfhydryl group compounds causing carcinogenesis in Gallbladder Cancer
  Protein ligand interaction between human hemoglobin chain A and chain B were studied by molecular docking where arsenous acid docked with human hemoglobin molecules and H-bonded with Cys104 and His 103 residues in chain A and His146 and Cys 93 residues in chain B Fig. 5A,B. The molecular interaction analyzed by ligplot has been presented in Fig. 5C for chain A and Fig. 5D for chain B which also shows the importance of SER 35 in the interaction. Interestingly, sulfhydryl group of taurine also interact with arsenic compound (Fig. 5D,E).

### Geological aspect

**Figure 6:**
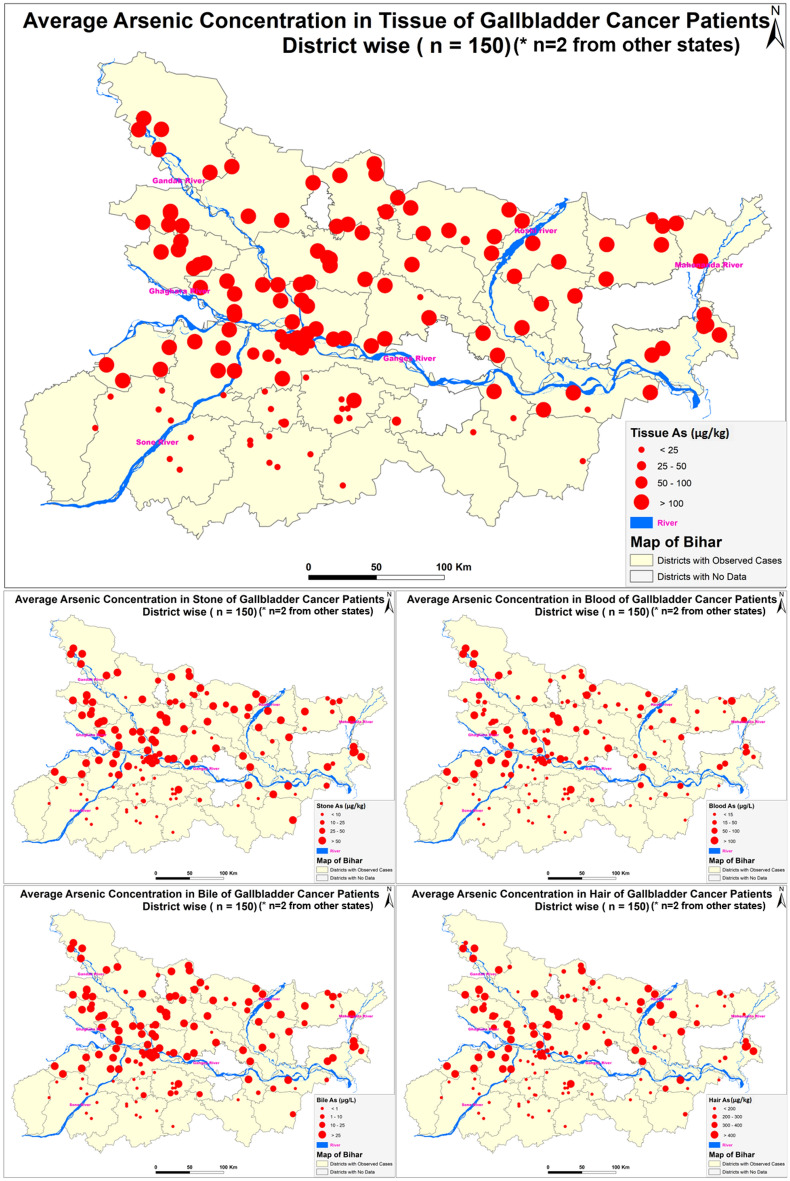
Map showing studied Gallbladder cancer patients with arsenic concentration in different biological samples with geomapping (Arc-Gis-10.5.1).

In 18 out of 38 districts in Bihar, have been affected by arsenic contamination in groundwater. Major source of arsenic for human consumption is through arsenic contaminated water^62^. The predominance of gall bladder cases is clearly brought out in the arsenic affected districts of Bihar (Fig. 6). The source of arsenic is primarily geogenic as it is acquiring endemic proportions and affecting a large geographical area. The rivers originating from extra peninsular region contain sediments with high arsenic content. The rivers carry these sediments which under favourable conditions releases arsenic in groundwater. The residency time, litho framework of the recharge vis a vis discharge area, age of the water in the affected aquifer and climatic conditions also have a role to play in enhanced contamination. The secondary enrichment in favourable conditions of oxic and anoxic waters supplemented by the geomorphological components also play a decisive role in increased contamination of aquifers at various depth levels. Arsenic contaminated aquifers are mainly present in newer alluvium (Holocene age) which is also supported by a model which defines the primary and secondary source.

## Discussion

Arsenic poisoning through drinking water and irrigation water in the recent times has caused serious health hazards in the population worldwide. Prolonged exposure to arsenic leads to many health outcomes. The diseases caused by this long-term arsenic exposure include skin manifestations, hypertension, ischemia, general body weakness, vascular disorders, auto-immune disorders (diabetes, arthritis, leukoderma), severe arteriosclerosis, neuropathies and many types of cancer^51,61,63^. In most of the cases if these symptoms are ignored, arsenic poisoning may lead to many types of cancer. One of highly correlated health outcome of arsenic poisoning is gallbladder cancer. In recent studies gallbladder cancer has been diagnosed in many patients coming from the arsenic hotspots of Gangetic plains of Bihar^64^. However, the etiology of aforementioned diseases leading to carcinogenesis has not been studied extensively. The exposure rate is so much high that one or more members in each exposed households exhibit arsenicosis symptoms^65–67^.

Arsenic has been classified as a class I human carcinogen by International Agency of Research on Cancer (IARC), which denotes that arsenic possesses the property of not only changing the configuration of the cellular activity but also modifying the gene functions at the genome level in human beings. In the recent studies, skin and several types of internal cancers such as bladder, kidney, liver, prostate and lung have been associated with arsenic ingestion^51,68–71^. Arsenic usually affects the cellular activities mainly by two pathways –arsenic induced oxidative stress and also through epigenetic changes. The former is mediated by the biotransformation of arsenic leading to the cellular damage through the production of reactive oxygen species (ROS). The ROS typically consists of superoxide anions, hydrogen peroxides, and hydroxyl radicals, which can damage the DNA causing chromosomal aberrations. Enhanced arsenic induced production of ROS by the cells has been linked to the carcinogenicity of arsenic^30,72–77^. The other suggested mechanism of arsenic carcinogenicity is the epigenetic changes which occur due to the changes in DNA methylation, histone modification and microRNAs. The hypermethylation of aberrant DNA promoter is strongly linked with the silencing of the tumour suppressor genes such as gene *p53*. The gene *p53* is known to be the guardian of the genome, and silencing of this important gene can lead to the transformation of a normal cell into a cancer cell^78–81^. The modification of histone proteins by histone acylation is also known to be the cause of arsenic carcinogenicity. Arsenic metabolites have been shown to modulate the normal histone patterns in the cells^82,83^. The microRNAs are small noncoding RNAs that inhibit the expression of the genes, and they can also induce carcinogenicity by deregulating the normal functions of the cell. The chronic exposure of As^III^ has been suggested to induce malignant transformation^84–87^. Gallbladder cancer in recent time has increased many folds in the Gangetic belt of India. The specific reasons for this increase have not been established yet, but few studies indicated the toxic metals or metalloids and pesticides as the causative agents^88–94^.

In the present study, consistently very high arsenic concentrations were recorded in the blood, gallbladder tissue, gallstones, gallbladder bile and the hair samples of gallbladder cancer patients. The comparison of the control (n = 200) and confirmed gallbladder cancer patients (n = 152) showed novel and significant findings. The study also confirms that the studied control subjects had non-significant arsenic concentration in their blood and hair samples.A significantly high arsenic concentration (*p* < 0.05) was detected in the blood samples with maximum concentration 389 µg/L in GBC cases in comparison to control. Similarly, in the gallbladder cancer patients, there was significantly high arsenic concentration observed in gallbladder tissue with highest concentration of 2166 µg/kg, in gallbladder stones 635 µg/kg, in bile samples 483 µg/L and in hair samples 6980 µg/kg respectively. There has been no benchmark range setup for the arsenic contamination in gall bladder tissue, bile, stones and blood, but for hair samples the normal levels of arsenic contamination in the unexposed human populaces ranges between 20 and 200 µg/kg. There was a significant positive correlation as increasing trend between the arsenic concentrations in between the gallbladder tissue, gallstone, bile, blood and hair samples of confirmed GBC patients were observed. Moreover, the n = 512 gallbladder cancer patient’s blood samples study revealed very significant arsenic concentration in the population of Bihar with maximum arsenic concentration as 746 µg/L. Out of n = 512, n = 350 blood samples had relatively had arsenic concentration more than 2 µg/L. This means that 68% of the studied population had significant arsenic concentration in their blood. Most of the patients were form the Gangetic plain region in comparison to the non-Gangetic plain area. This positive trend indicates a strong association between arsenic contamination and the observed carcinogenesis.

No signalling pathways have yet found linking arsenic accumulation in the gallbladder tissue or gallstones or gallbladder bile to carcinogenicity. Nonetheless, the present study provides strong circumstantial evidence associating arsenic accumulation with the progression of the gallbladder carcinogenesis. Arsenic in the Gangetic plains of Bihar is also found in the form of arsenopyrite (FeAsS)^95^. It has very high affinity to bind with these two elements, hence it easily binds with endogenous sulfhydryl compounds including metallothioneins. Arsenic reaches the human blood after the gastrointestinal absorption and may cause gallbladder carcinogenesis in two ways. First, it is hypothesized that, arsenic binds with RBC since haemoglobin in RBC contains the iron, and then after the completion of RBC lifespan (120 days) reaches liver via spleen^96–98^. In liver, arsenic binds with cysteine residues which compounds with sulfhydryl group and accumulates further^99–103^. Subsequently, arsenic binds with the next sulfhydryl group containing compound taurine^104–111^. Taurine is the key factor which controls the formation of the gallstones, however arsenic likely supresses its function by lowering its levels. This initiates the formation of gallstones with sludge in the gallbladder due to the precipitation of the cholesterol^112,113^. The taurine has the conjugation with the chenodeoxycholic acid (CDCA), that is why high arsenic contamination was observed in the bile of the GBC patients. Gallstones in due course of time causes inflammation in the inner wall of the gallbladder tissue, which after mutations transforms into a cancerous tissue^113^. Secondly, it is also hypothesized that arsenic in the gallbladder elicits rigorous production of ROS, leading to frequent mutations in the genes p53, p21, p16 and KRAS. Such mutations in turn induces inflammation in the epithelial cells of gallbladder causing conversion of normal cells to dysplasia, which in the long run leads to gallbladder carcinoma^112,114–131^.

Molecular Docking studies of arsenic compounds with haemoglobin alpha chain and beta chain have revealed the efficient binding of arsenic compounds with cysteine residues. Arsenous acid was able to bind with Cys 104 residue of haemoglobin alpha chain globin chain A and also binds with the Cys 93 residue of haemoglobin beta chain globin chain B. Other important residues which can be involved in arsenic toxicity to haemoglobin are His, Ser which are efficiently involved in the interaction with arsenic. The toxicity of trivalent arsenicals likely to occur through the interaction of trivalent arsenic species with sulfhydryl groups in proteins. Trivalent arsenicals have high affinity for sulfhydryl groups and can bind to reduced cysteines in peptides and proteins. Several studies have reported the binding of trivalent arsenicals to the cysteine residues of proteins such as iAsIII binds at three cysteine residues, Cys32, Cys34 and Cys37 of ArsR repressor protein of *E. coli*. Cys156 and Cys206 of Arsenic methyltransferase (AS3MT) of human are the active site for arsenic binding whereas Cys72, Cys174 and Cys224 of AS3MT of thermophilic eukaryotic red algae *Cyanidioschyzon merolae* are believed to be involved in arsenic binding. Hence, the present study also strongly indicates strong association between the chronic arsenic contamination in the Gangetic plains of the Bihar (India) and gallbladder carcinogenesis.

A previous significant study also reported association between the arsenic exposure and gallbladder cancer incidences in the populations of India and Japan. However, the study was conducted using a very small number of gallbladder cancer patients (8 Indian and 5 Japanese GBC cases), and therefore a strong correlation could not be made. Moreover, they also suggested association of gallbladder carcinogenesis with the other heavy metals like chromium, cadmium, lead, zinc and mercury^132^.

Similarly, in Chile (South America), the annual cancer incidences are approximately, 35,000 new cases per year and the cancer burden in the population in recent times has increased many folds similar to India^133–136^. The gallbladder cancer incidences in Chile have a higher ratio in comparison to the average data on a global scale^137,138^. Unfortunately, Chile also reports high arsenic concentration in the groundwater with concentrations as high as about 5,000 µg/L^139^. Not much is known about the association between arsenic exposure and increased gallbladder cancer cases in Chile^140,141^. Recent studies, reported that if the exposed subjects are consuming arsenic contaminated water for long duration may cause risk of gallbladder cancer^142–144^.

The PCA results demonstrated that the internal arsenic concentrations could not characterize all the severe gallbladder patients, but all the highly arsenic-loaded subjects were the severe gallbladder patients. This suggests the association between arsenic intake and gallbladder cancer. The high internal arsenic concentration may cause accelerating the progress of gallbladder cancer. Another suggestion was that a single biological medium might not be sufficient to understand the real affection of the internal arsenic concentrations: e.g., some patients had high arsenic concentrations in gallbladder tissues, but hair samples showed low concentrations and vice versa. The way how arsenic is metabolized and accumulated in bodies may differ depending on the individuals. Moreover, the arsenic binding to cysteine of RBC, taurine in liver is well proved through our molecular docking which denotes the pathway of GBC disease carcinogenesis. This novel pathway of disease carcinogenesis strongly validates the hypothesis.

## Conclusions

From the entire study, it can be concluded from the present study that gallbladder cancer is prevalent in the Gangetic plains of Bihar, where the arsenic contamination in the exposed population is very high increasing the disease burden of the state. The significantly high arsenic concentration observed in the gallbladder tissue, gallbladder stone, bile, blood and hair samples in GBC patients strongly indicates the linkage between high chronic exposure to arsenic and gallbladder carcinogenesis. The GBC disease burden is significantly increasing manyfolds in the arsenic exposed population. However, the other confounding factors can also add the disease burden of GBC manifolds. The novel pathway of GBC carcinogenesis validates that arsenic is one of the important toxicants which is responsible for causing the disease in this particular area. Hence, there is an urgent need to control the disease burden by developing policies and guidelines that will alleviate arsenic exposure to the impacted populations.

## Supplementary Information

Supplementary Information.

## Data Availability

The datasets used for the bioinformatic analysis is publicly available from RCSB Protein Data Bank (https://www.rcsb.org/). The Human Haemoglobin Molecule 3D -X-ray Crystallographic structure at PDB (PDB ID:4HHB) is freely accessible at https://www.rcsb.org/structure/4HHB (https://doi.org/10.2210/pdb4HHB/pdb). The software used in the map layer generation was ArcGIS Version 10.5.1. All the other data materials have been shared in the Zip form in the journal which on request can be obtained from the journal.

## Abbreviations

GBC: Gall bladder cancer
ICMR: Indian Council of Medical Research
MMA: Monomethylarsonous acid
DMA: Dimethylarsinic acid
TMA: Trimethylarsine
ROS: Reactive oxygen species
USEPA: U.S Environmental Protection Agency
WHO: World Health Organisation
GF-AAS: Graphite Furnace Atomic Absorption Spectrophotometer
PCA: Principal component analysis
