# Supplementary material for: Arsenic causing gallbladder cancer disease in Bihar
Source: Sci Rep. 2023 Mar 14;13:4259. doi: 10.1038/s41598-023-30898-0 (PMC10014949; doi:10.1038/s41598-023-30898-0)
Supplement: Supplementary file 1 — Supplementary Information. [file 41598_2023_30898_MOESM1_ESM.docx]

**SAMPLE SIZE CALCULATION**

**n = Z^2^ *P*(1-*P*)/d^2^**

where *n* = sample size,

*Z*= Z statistic for a level of confidence,

*P* = expected prevalence or proportion

(in proportion of one; if 20%, *P*=0.2), and

*d* = precision

(in proportion of one; if 5%, *d*=0.05).

*Z* = 1.96 (for 95% confidence intervals (CI))

*P* = 0.11 (for 11% prevalence)

*d* = 0.05 (precision (in proportion of one, if 5%)

***Ref: Daniel WW. Biostatistics: A Foundation for Analysis in the Health Sciences. 7^th^ edition. New York: John Wiley & Sons); 1999.***
